# Pitch and Rhythm Perception and Verbal Short-Term Memory in Acute Traumatic Brain Injury

**DOI:** 10.3390/brainsci11091173

**Published:** 2021-09-03

**Authors:** Kirsten S. Anderson, Nathalie Gosselin, Abbas F. Sadikot, Maude Laguë-Beauvais, Esther S. H. Kang, Alexandra E. Fogarty, Judith Marcoux, Jehane Dagher, Elaine de Guise

**Affiliations:** 1 Psychology Department, University of Montreal, Montreal, QC H2V 2S9, Canada; nathalie.gosselin@umontreal.ca; 2 Centre de Recherche Interdisciplinaire en Réadaptation du Montréal Métropolitain (CRIR), Montreal, QC H3S 1M9, Canada; jehane.dagher@mcgill.ca; 3 International Laboratory for Brain, Music and Sound Research (BRAMS), and Centre for Research on Brain, Language, and Music (CRBLM), Montreal, QC H2V2S9, Canada; 4 Neurology and Neurosurgery Department, McGill University Health Centre, Montreal, QC H4A 3J1, Canada; abbas.sadikot@mcgill.ca (A.F.S.); maude.lague-beauvais@muhc.mcgill.ca (M.L.-B.); judith.marcoux@mcgill.ca (J.M.); 5 Traumatic Brain Injury Program, McGill University Health Centre, Montreal, QC H3G 1A4, Canada; 6 Faculty of Medicine, McGill University, Montreal, QC H3G 2M1, Canada; shin.h.kang@mail.mcgill.ca; 7 Department of Neurology, Division of Physical Medicine and Rehabilitation, Washington University School of Medicine, St. Louis, MO 63110, USA; afogarty@wustl.edu; 8 Research Institute of the McGill University Health Centre, Montreal, QC H4A 3J1, Canada

**Keywords:** traumatic brain injury, verbal short-term memory, music perception, pitch, rhythm

## Abstract

Music perception deficits are common following acquired brain injury due to stroke, epilepsy surgeries, and aneurysmal clipping. Few studies have examined these deficits following traumatic brain injury (TBI), resulting in an under-diagnosis in this population. We aimed to (1) compare TBI patients to controls on pitch and rhythm perception during the acute phase; (2) determine whether pitch and rhythm perception disorders co-occur; (3) examine lateralization of injury in the context of pitch and rhythm perception; and (4) determine the relationship between verbal short-term memory (STM) and pitch and rhythm perception. Music perception was examined using the Scale and Rhythm tests of the Montreal Battery of Evaluation of Amusia, in association with CT scans to identify lesion laterality. Verbal short-term memory was examined using Digit Span Forward. TBI patients had greater impairment than controls, with 43% demonstrating deficits in pitch perception, and 40% in rhythm perception. Deficits were greater with right hemisphere damage than left. Pitch and rhythm deficits co-occurred 31% of the time, suggesting partly dissociable networks. There was a dissociation between performance on verbal STM and pitch and rhythm perception 39 to 42% of the time (respectively), with most individuals (92%) demonstrating intact verbal STM, with impaired pitch or rhythm perception. The clinical implications of music perception deficits following TBI are discussed.

## 1. Introduction

Amusia is a disorder in music processing that is independent of musical training, and may be present despite normal intelligence, verbal memory skills, language skills, and auditory functioning [1,2]. It is estimated that between 1.5% and 4.2% of the general population has congenital amusia [3]. Music perception deficits after acquired brain injury and neurosurgical interventions are often referred to in the literature as acquired amusia. Whereas acquired music perception deficits have been studied in the context of stroke [4,5,6,7,8,9,10], surgical interventions following epilepsy [10,11,12,13,14], and the clipping of aneurysms [15,16,17], few studies have examined deficits in music perception following traumatic brain injury (TBI) [18,19,20]. Although patients with head injury may show evidence of acquired amusia to various degrees, these deficits have not been studied using rigorous quantitative measures integrated with brain imaging findings. 

The majority of amusia studies examine musical pitch processing. Thus, amusia is often attributed to difficulty processing pitch, whether related to pitch-specific memory deficits [21,22,23,24,25,26], or pitch-specific perceptual deficits [2,27]. Deficits in pitch perception can interfere with rhythm discrimination [28], and the ability to accurately perceive durations between notes [29]. This may be due to simultaneous demands made on pitch and rhythm processing. However, evidence indicates that pitch deficits in amusia may be dissociable from rhythm deficits [8,30,31,32]. The Montreal Battery for the Evaluation of Amusia (MBEA) is the most widely-used measure to diagnose perceptual deficits [33], with subtests comprising both the melodic dimension (sequential variations in pitch) and temporal dimension (sequential variations in duration) [2]_._ Given the MBEA’s usefulness in identifying music disorders following brain damage, the TBI population provides a novel opportunity to determine how performance on the MBEA relates to the lateralization of pitch and rhythm perception. 

### 1.1. Neural Networks Underlying Pitch and Rhythm Discrimination

Individuals with congenital amusia demonstrate pitch deficits associated with abnormalities in a predominantly right frontotemporal network [25,26,34,35,36]. This is evidenced in increased grey matter density and decreased white matter density in the right inferior frontal gyrus [37], and right superior temporal gyrus [38], compared with controls [25,27]. The volume of the right arcuate fasciculus is reduced [27,39,40]. In addition, there is diminished connectivity between the right auditory cortex and the right inferior frontal gyrus [25,27,39], with anomalous recurrent processing between these regions [27,39]. Finally, there is increased activity between the left and right auditory cortices [25]. In sum, congenital amusia is associated with decreased activity between inferior frontal and auditory regions, and increased activity between auditory cortices [27,41].

Overlapping neural networks appear to underlie pitch and rhythm processing [42], and pitch and rhythm disorders may co-occur in individuals with congenital amusia [19,43]. However, several imaging studies demonstrate that pitch and rhythm processing are at least partly dissociable [8,30,31,32,42,44], with more widespread neural activation occurring during rhythm processing [45]. For example, a PET study of healthy individuals demonstrated that both pitch and rhythm activated a frontoparietotemporal network. However, only rhythm processing recruited the subcortical region of the cerebellum [42]. Indeed, rhythm involves activation of both cortical (i.e. prefrontal cortex, premotor cortex, supplementary motor area, temporal lobe, and parietal cortex), and subcortical (i.e. cerebellum, and basal ganglia) areas [45,46,47]. Therefore, either damage to a predominantly right frontoparietotemporal network or damage to subcortical areas can result in impaired interval timing and duration estimation [48]. In summary, studies of pitch and rhythm indicate involvement of diffuse brain regions during musical processing, with most studies indicating right-hemisphere anomalies.

### 1.2. Acquired Amusia in Individuals with Traumatic Brain Injury and Stroke 

Traumatic brain injury may be defined as “an alteration in brain function, or other evidence of brain pathology, caused by an external force” [49], and may result in disruption of attention, memory, and executive functions [50]. Given the extensive cortico-cortical networks underlying amusia, TBI patients may have a higher incidence of music perception deficits than that observed in the general population. Indeed, a study conducted by Balzani and colleagues (2014) reported that mild TBI patients demonstrated impaired performance on the MBEA Rhythm test (*p* = 0.01) and a trend in poorer performance on the MBEA Scale test (*p* = 0.07) than controls [18]. A recent study confirmed that TBI patients performed significantly worse than healthy controls on both the Scale and Rhythm tests of the MBEA [19]. Both studies evaluated TBI patients following a significant time lapse, from one to 10 years post-injury. Neither study evaluated regional abnormalities on brain imaging in TBI patients, relative to deficits on the Scale and Rhythm subtests of the MBEA. Furthermore, music perception was not examined in TBI patients during the acute period of recovery, defined here as the first three months following injury. Thus, to our knowledge, the present study is the first to examine and relate regional abnormalities on brain imaging to music perception in patients during the acute phase of TBI, when music interventions are often initiated.

Studies conducted in stroke populations report impaired performance on the Scale test, particularly in cases of right hemisphere damage [6,9,36]. For example, among patients with middle cerebral artery damage, those with right hemisphere damage have more severe pitch deficits on the MBEA than those with left hemisphere damage [36]. This is particularly true for patients with damage to frontal and temporal regions [36]. Together, these studies demonstrate that individuals with brain damage have poorer performance on the Scale and Rhythm tests than do healthy individuals. 

### 1.3. The Question of Modularity in Short-Term Memory for Rhythm and Pitch 

The frontotemporoparietal network that is associated with amusia has also been associated with domain-general short-term memory (STM) [36,51]. Short-term memory is defined here as the maintenance of stored auditory or verbal information for seconds to minutes, without manipulation [52,53]. Therefore, an important question arises as to whether domain-general STM deficits underlie amusia, or whether a pitch-specific memory deficit underlies amusia [34]. Several studies provide evidence that verbal memory and pitch memory are shared domains [6,24,36,54,55,56]. Indeed, a recent meta-analysis concluded that trained musicians perform significantly better at both verbal and tonal short-term memory tasks than non-musicians [57]. The fact that musical training is associated with greater verbal STM points to shared mechanisms between musical STM and verbal STM. Indeed, one study conducted in stroke patients found that acquired amusia was associated with deficits in domain-general attention, executive functions, and working memory [6]. 

However, other studies have proposed separate memory systems for pitch and verbal material [22,58,59,60]. Evidence for this has been demonstrated in healthy participants, who are impaired at recognizing and maintaining tones in STM when there is interference of irrelevant tones, but not words [58,60]. Studies in congenital amusia also propose that STM for pitch and verbal STM are dissociable [22,34], based on findings of spared verbal STM, with impaired pitch memory [1,23,61]. It is as yet unclear whether short-term memory mechanisms for music and verbal material are shared, overlapping, or separate. However, a recent fMRI study conducted in congenital amusics proposes that music perception deficits may be explained by an inability to integrate higher order processing, such as STM, with the elemental components of music [34]. Research in TBI patients, who commonly suffer attention and memory deficits, might further elucidate the mechanisms underlying music processing, and deficits associated with amusia.

### 1.4. Goals of the Present Study

We aimed (1) to evaluate the performance of mild to severe patients with TBI on pitch and rhythm processing during the acute phase of injury, compared with healthy controls. We hypothesized that a TBI patient population would demonstrate impaired performance on music perception tasks compared to healthy controls; (2) to determine and characterize the possible co-occurrence of pitch and rhythm disorders in TBI patients. We hypothesized that pitch and rhythm perception deficits would co-occur in TBI patients, who often have diffuse injury; (3) to determine in a TBI population whether damage predominantly located in the right or left hemisphere is associated with the hemispheric lateralization of pitch and rhythm discrimination. Based on studies conducted in individuals with congenital amusia and acquired amusia following stroke, we hypothesized that TBI patients with right hemisphere damage would have greater deficits than those with left hemisphere damage; and (4) to determine if verbal short-term memory is related to pitch and rhythm perception in TBI patients, who often have STM deficits. Given the fact that previous studies argue for at least partly overlapping mechanisms of verbal STM and pitch STM, we hypothesized that TBI patients with lower scores on MBEA tasks would have lower scores on a verbal short-term memory task.

## 2. Materials and Methods

### 2.1. Participants

Forty-two mild, moderate, and severe TBI in-patients between the ages of 20 and 80 who were hospitalized on the neurology and neurosurgery ward at the McGill University Health Centre-Montreal General Hospital (MUHC-MGH) were included in this study (Table 1). Patients seen only in the emergency room were not included. 

TBI patients who participated had an initial Glasgow Coma Scale test score of 3–15, and were admitted to our tertiary traumatology center. Scores from arrival, emergency, and post-resuscitation were collected by the research assistant from the patient’s medical chart. A GCS score between 3 and 8 was considered to be a severe TBI, a score between 9 and 12 was considered moderate, and a score between 13 and 15 was considered mild [62,63]. Within the mild TBI population is a subgroup referred to as “mild complex”, a radiological diagnosis applied to patients with positive findings on CT. The TBI diagnosis was confirmed by a physician, based on the Centers for Disease Control and Prevention criteria [64]. Patients who were included in the sample had sustained a closed head injury, meaning that the there was no penetrating injury. Therefore, direct or indirect force (rotational and/or deceleration) to the head caused the injury.

Exclusion criteria for the study included a pre-morbid history of alcohol or drug abuse, an active diagnosis of psychiatric disorder, pre-existing neurological deficits, post-traumatic agitation precluding collaboration, aphasia, inner ear fractures, hemotympanum, and visual and hearing impairment. Hearing impairment was determined by asking each participant if they had suffered a hearing loss or had a history of congenital impairment, in addition to verifying with each individual that they were capable of hearing the sample musical excerpts with ease during practice trials. Assessments were not performed when patients were administered intravenous narcotic medication, or while they were still in the intensive care unit. Patients not fluent in English or French were excluded, as well as those who were unable to consent. To compare the performance on music perception tests, the TBI patients were matched for age and education with 42 healthy control participants selected from the norms of 421 participants who completed the MBEA (Peretz, et. al., 2003; see the Isabelle Peretz Research Laboratory website, https://www.peretzlab.ca/publications/2003/page2, accessed on 25 March 2020). Matching was done based on age and education level only, while remaining blind to the scores of each individual. TBI patients and control groups were not equivalent with respect to sex, *X*^2^ (1, *N* = 84) = 11.23, *p* < 0.00.

### 2.2. Measures and Procedure

Demographic, medical, accident-related and musical training characteristics were retrieved from each patient’s medical file, and collected through questions during their hospitalization (Table 1). Consent to participate in the study was obtained when the patient’s condition was medically stable, following resolution of post-traumatic amnesia. All evaluations were conducted by research assistants who were trained by a neuropsychologist. Extended Glasgow Outcome Scale assessments were done by the treatment team upon discharge from hospital [65]. The Scale and Rhythm subtests of the MBEA along with a verbal short-term memory test were administered at each patient’s bedside for a total duration of 25–30 min, with breaks between subtests as needed.

#### 2.2.1. Scale and Rhythm Tests of the Montreal Battery of Evaluation of Amusia (MBEA)

These two subtests of musical perception were administered to evaluate whether the TBI patients had deficits following their injuries, using the total score for each subtest. The Rhythm test and Scale test are thought to tap relatively distinct abilities or processing components, with the Scale test measuring musical perception on the melodic dimension (varying sequences of pitch that are thought to be processed by a subsystem that specifies melodic contour and tonal functions), and the Rhythm test measuring musical perception on the temporal dimension (varying sequences of duration thought to be processed by a relatively independent subsystem in parallel to the processing of the melodic dimension; this subsystem treats the rhythmic structure and metric organization of music) [2]. The Scale and Rhythm subtests of the MBEA were chosen (1) to facilitate comparison with previous studies in chronic TBI patients, and (2) because acute TBI patients are easily fatigued and would likely have difficulty completing the entire MBEA battery. The Scale and Rhythm subtests were played at the patient’s bedside through a wireless Bose SoundLink Color Bluetooth speaker II (Bose Corporation, Framingham, MA, USA) during a time in which patients were uninterrupted. The order of tests was counterbalanced across participants.

Each test lasted 10 min, and consisted of two practice trials, followed by 30 trials, and one catch trial. Feedback was limited to the practice trials. Each trial began with a warning tone, after which the patient was asked to compare a pair of musical stimuli for sameness. First, there was a target stimulus, followed by two seconds of silence, and then a comparison stimulus. There was an inter-stimulus interval of five seconds. In half of the trials, the Scale test contained one pitch that was altered to be out of scale, without changing the contour of the melody. 

The Rhythm test consisted of the same melodies as the Scale test. However, in half of the trials, the rhythmic grouping of two of the tones was altered, while the meter and total number of sounds remained identical. 

The MBEA has good sensitivity, with fewer than 80% of the participants not achieving perfect scores on the subtests. Test-retest reliability is also adequate (*r* = 0.75, *p* < 0.01), as is convergent validity, when compared with Gordon’s Musical Aptitude Profile (*r* = 0.53, *p* < 0.001). Furthermore, it has been demonstrated to be useful in identifying music perception difficulties in populations with brain insult, such as stroke, resection of tissue in epilepsy, and the surgical clipping of an aneurysm [2]. 

#### 2.2.2. Digit Span

This is a subtest of the Wechsler Memory Scale (WMS-III) [66]. Digit Span Forward is thought to measure short-term memory, or temporary storage of information. Patients were asked to repeat a sequence of digits in order that had been verbally presented by the research assistant, with the longest span calculated. The Digit Span has an internal consistency of α = 0.94 and a test re-test reliability of *r* = 0.74 [67]. 

#### 2.2.3. Extended Glasgow Outcome Scale (GOSE)

This widely-used scale measures functional outcomes after acquired brain injury by classifying patients into eight broad outcome categories, including death, vegetative state, lower severe disability, upper severe disability, lower moderate disability, upper moderate disability, lower good recovery, and upper good recovery [68].

### 2.3. Image Acquisition

Computerized tomography (CT) scans were acquired from the radiology department of the Montreal General Hospital using a Toshiba scanner (Minato City, Tokyo, Japan). All CT images were reconstructed to 2.5 mm thick slices acquired in an axial orientation. Patients were scanned at the discretion of the attending emergency department physician, as per Canadian CT Scan guidelines [69], and each scan was independently reviewed by a neurosurgeon certified under the Royal College of Physicians and Surgeons of Canada. 

### 2.4. Statistical Analyses

1. To evaluate the performance of TBI patients versus controls on pitch and rhythm processing, a quasi-experimental, paired-samples t-test design was used in a sample of TBI patients who were age- and education- matched with normal controls as independent variables, and the Scale test and Rhythm test total scores as dependent variables. An alpha level of 0.05 was used for all analyses.
2. To determine whether there was a relationship between performance on the Scale and Rhythm tests of the MBEA, Pearson product-moment correlation coefficients were calculated.
3. To determine whether damage predominantly located in the right or left hemisphere was associated with the hemispheric lateralization of pitch and rhythm discrimination, two 2-tailed independent samples t-tests were used to compare patients with left hemisphere damage to those with right hemisphere damage. In both, the independent variable was injury location, and the dependent variables were total score on the Scale test and the Rhythm test.
4. To determine whether there was a relationship between performance on the Scale and Rhythm tests, and severity of brain injury, Pearson product-moment correlation coefficients were calculated.
5. To determine associations between performance on the Scale and Rhythm tests and verbal short-term memory tests (*Z* scores representing longest digit span forward), Pearson product-moment correlation coefficients were calculated.

All analyses were performed using SPSS 24.0.

## 3. Results

### 3.1. Data Integrity 

During testing, there were 11 randomly missing values for Digit Span Forward (*n* = 31), and 6 randomly missing CT scan values (*n* = 36). There were no missing values for the other measures. There were no outliers in the sample. In the Scale and Rhythm tests, all variables of interest were normally distributed, with skewness values below three, and kurtosis values below 10 [70]. 

### 3.2. Comparison of Music Perception Scores in TBI Patients versus Controls

#### 3.2.1. Scale Test

**Figure 1:**
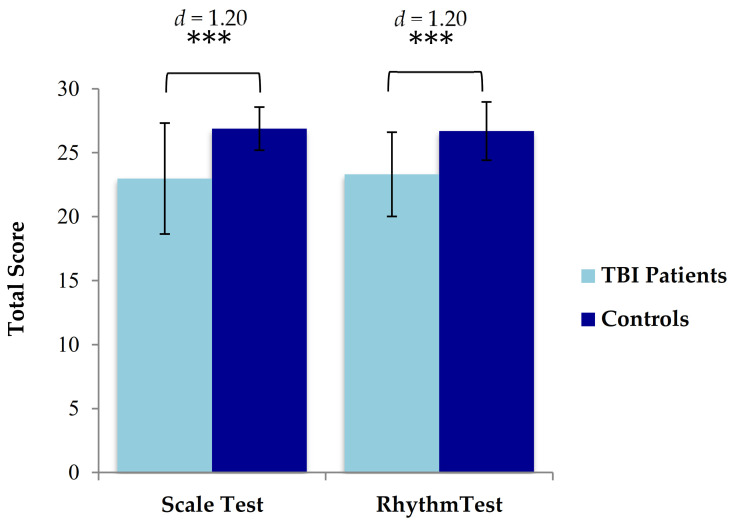
Mean scores and standard errors of TBI patients and matched controls. Controls performed significantly better than TBI patients on both the Scale test and the Rhythm Test. *** = *p* ≤ 0.001.

**Figure 2:**
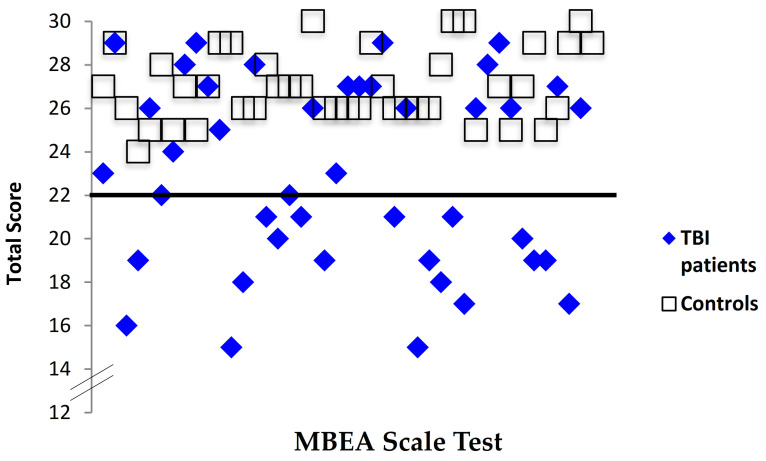
Individual scores for TBI patients and controls on the MBEA Scale test. Note. The black line represents the cutoff score for this test, which corresponds to two standard deviations below the mean (Peretz, 2003).

To test the hypothesis that TBI patients would have poorer perceptual skills compared with controls when judging musical input on the melodic dimension, the total score (/30) was calculated. TBI patients had significantly lower scores *(M* = 22.98, *SD* = 4.34) than controls *(M* = 26.88, *SD* = 1.70), indicating poorer pitch perception than that of controls, *t* (41) = −5.42, *p <* 0.001 (Figure 1). Forty-three percent of TBI patients (18/42) had Scale test scores below the cut-off of 22 as indicated in the MBEA [2], compared with none of the healthy controls (0/42), indicating a deficit in pitch perception (Figure 2). 

#### 3.2.2. Rhythm Test

**Figure 3:**
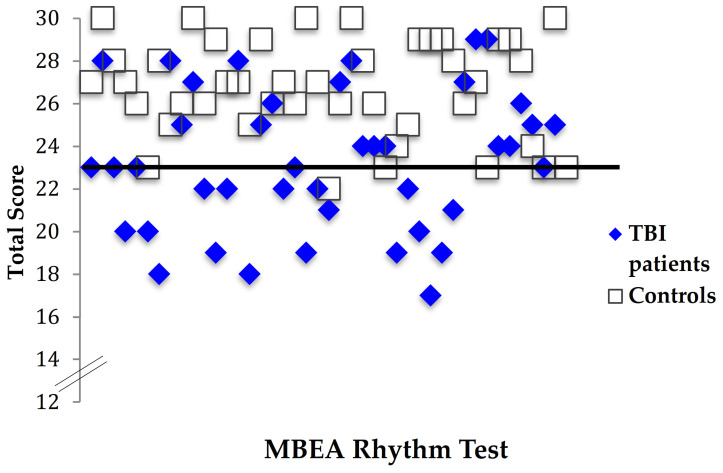
Individual scores for TBI patients and controls on the MBEA Rhythm test. Note. The black line represents the cutoff score for this test, which corresponds to two standard deviations below the mean (Peretz, 2003).

To test the hypothesis that TBI patients have poorer perceptual skills compared with controls when judging musical input on the temporal dimension, the total Rhythm test score was calculated, and a two-tailed paired samples *t*-test conducted. TBI patients had significantly lower scores (*M* = 23.31, *SD* = 3.29) than controls (*M* = 26.69, *SD* = 2.28), indicating lower perceptual ability for rhythm than that of controls, *t* (41) = −5.43, *p * < 0.001 (Figure 1). Forty percent of the TBI patients (17/42) had Rhythm test total scores that fell below the cut-off of 23 as indicated in the MBEA [2], compared with only one of the healthy controls (1/42), indicating a deficit in rhythm perception (Figure 3). All patients and controls passed the catch trial for both subtests [2].

### 3.3. The Co-Occurrence of Pitch and Rhythm Deficits in Acquired Amusia 

A moderate positive correlation was found between performance on the Scale test and the Rhythm test, *r* (40) 0.61, *p* ≤ 0.001. Thirty-one percent of the TBI patients (13/42) had co-occurring pitch and rhythm deficits (Table 2). In addition, nine patients had deficits exclusively in pitch, and two patients had deficits exclusively in rhythm. 

### 3.4. Neuroimaging and Performance on the Scale and Rhythm Tests of the MBEA

Of the 36 patients who had CT scans performed, 18 demonstrated trauma-related brain abnormalities. Of these, two were classified according to the GCS score as severe, seven were moderate, and nine were mild complex. Group sizes were comparable with respect to lateralization (right, *n* = 6; left, *n* = 7; bilateral, *n* = 5)(Table 2). The data were normally distributed among sub-groups, and parametric tests were applied. 

An independent samples t-test was applied to compare performance on the Scale test in TBI patients with injury involving the right hemisphere (right, bilateral) (*M* = 20.36, *SD* = 3.93) to those without injury involving the right hemisphere (left, no mass effect) (*M* = 23.92, *SD* = 4.13), *t* (34) = −2.68, *p* = 0.011, *d* = 0.97, indicating that damage to the right hemisphere results in decreased pitch perception.

Performance on the Rhythm test was compared in TBI patients with injury involving the right hemisphere (right, bilateral) (*M* = 21.18, *SD* = 2.64) versus those without right hemisphere damage (left, no mass effect) (*M* = 24.04, *SD* = 3.45). Patients with right hemisphere damage performed worse on the Rhythm test, *t* (35) = −2.45, *p* = 0.020, *d* = 0.93), indicating that damage to the right hemisphere decreases rhythm perception. 

**Figure 4:**
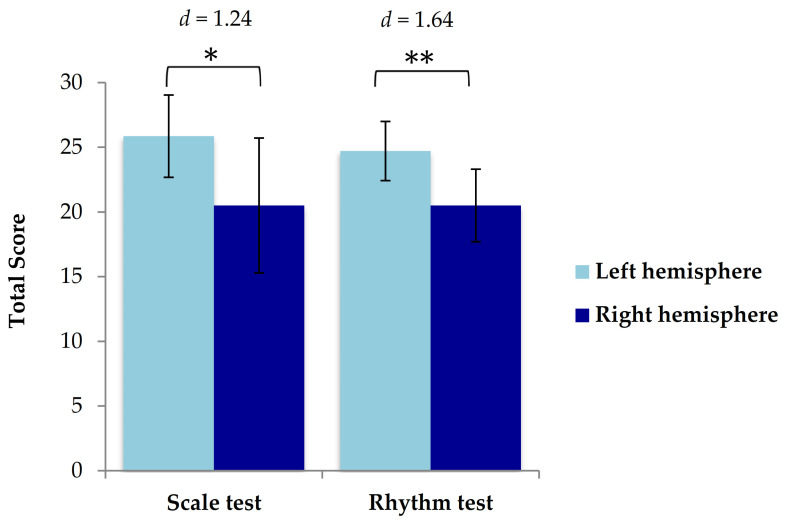
Mean values of total score in patients with injury located in the right hemisphere and left hemisphere on the Scale test and Rhythm test of the MBEA. Standard errors of the mean are represented by the error bar attached to each column. Patients with injury located in the right hemisphere performed more poorly on the Scale test than those with injury located in the left hemisphere.* = *p* ≤ 0.05, ** = *p* ≤ 0.01.

Finally, two-tailed independent samples *t*-tests were performed to determine whether TBI patients with right hemisphere damage (*n* = 6) would perform more poorly than those with left hemisphere damage (n = 7) when bilateral cases (*n* = 5) were excluded. Results on the Scale test indicated that patients with right hemisphere damage (*M* = 20.50, *SD* = 5.21) performed worse than those with left hemisphere damage (*M* = 25.86, *SD* = 3.18), *t* (11) = −2.28, *p* = 0.044, despite decreasing the sample size by excluding bilateral cases (Figure 4). 

Results on the Rhythm test indicated that patients with right hemisphere damage (*M* = 20.50, *SD* = 2.81) also performed more poorly than those with left hemisphere damage (*M* = 24.71, *SD* = 2.29), *t* (11) = −2.98, *p* = 0.012, again indicating that patients with right hemisphere damage had poorer music perception than patients with left hemisphere damage (Figure 4). 

### 3.5. Severity of Brain Injury and Music Perception Deficits

There was no correlation found between the GCS and the Scale test total score, *r* (40) = 0.12, *p =*.45, or the Rhythm test total score, *r* (40) = 0.26, *p* = 0.10.

### 3.6. Verbal Short-Term Memory and Music Perception Deficits

#### 3.6.1. Longest Digit Span Forward 

On the Digit Span Forward subtest, TBI patients scored in the low average range (*Z* = −1.01, *SD* = 0.67), with spans between four and seven digits, indicating a weakness in short-term memory. Three individuals had scores that were at least two standard deviations below average, indicating verbal STM deficits. See Table 2 for performance on a case-by-case basis.

#### 3.6.2. The Relationship between Music Perception Scores and Verbal Short-Term Memory

**Figure 5:**
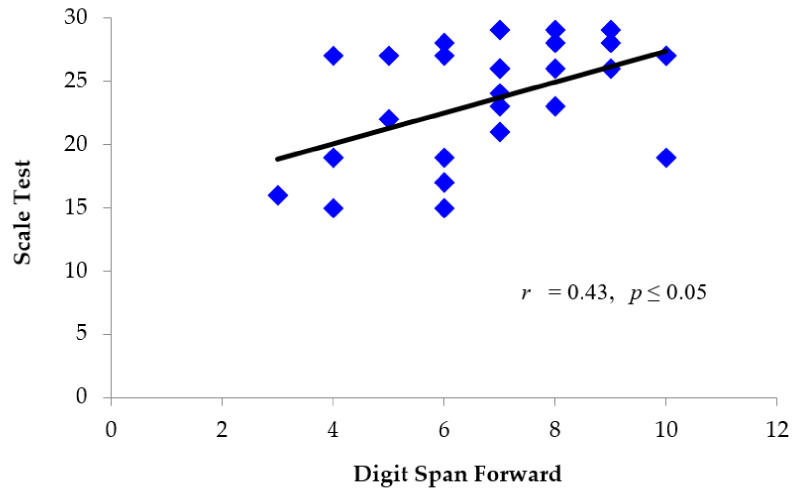
Individual scores for TBI patients depicting the relationship between performance on the MBEA Scale test (total score) and Digit Span Forward (scaled score). Patients who performed better on the Scale test also tended to perform better on Digit Span Forward.

**Figure 6:**
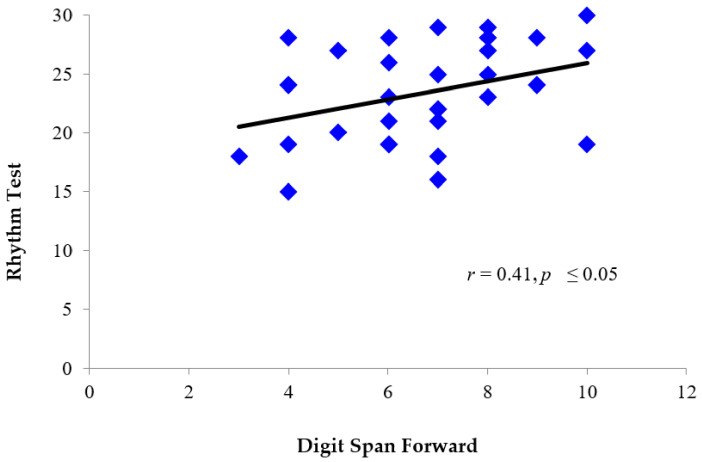
Individual scores for TBI patients depicting the relationship between performance on the MBEA Rhythm test (total score) and digit span forward (scaled score). Patients who performed better on the Rhythm test also tended to perform better on Digit Span Forward.

Pearson product-moment correlation coefficients were calculated to determine if there was a relationship between performance on the Scale and Rhythm tests, and performance on verbal short-term memory tasks. A moderate positive correlation was found between longest Digit Span Forward (*Z* = −0.90, *SD* = 0.79), and performance on both the Scale test (*M* = 23.19, *SD* = 4.62) *r* (29) = 0.43, *p* ≤ 0.05 (Figure 5), and Rhythm test (*M* = 23.30, *SD* = 3.89) *r* (29) = 0.41, *p* ≤ 0.05 (Figure 6). There was no significant difference in performance on Digit Span Forward in patients with right (*M* = -.87, *SD* = 0.62) versus left (*M* = 1.00, *SD* = 0.82) hemisphere damage *t* (10) = 0.30, *p* = 0.767.

#### 3.6.3. Pitch Processing and Verbal Short-Term Memory

Fifty-eight percent of the TBI patients demonstrated either intact performance on both the Scale test and Digit Span Forward, or impaired performance on these two tests (*n* =18). Of the 42% that demonstrated deficits exclusively on one test or the other (*n* = 13), 12/13 demonstrated intact performance on Digit Span Forward coupled with impaired performance on the Scale test (Table 2).

#### 3.6.4. Rhythm Processing and Verbal Short-Term Memory

Sixty-one percent of the TBI patients demonstrated intact performance on the Rhythm test and Digit Span Forward, or impaired performance on these two tests (*n* =19). Of the 39% that demonstrated deficits exclusively on the Rhythm test or on Digit Span Forward (*n* = 12), 10/12 demonstrated intact performance on Digit Span coupled with impaired performance on the Rhythm test (Table 2).

#### 3.6.5. Location of Injury, Music Perception Scores, and Verbal Short-Term Memory Scores 

Scans were available for 15 patients who completed the Scale and Rhythm tests along with Digit Span Forward. Of these, six patients with damage involving right frontal regions had impaired performance on the Scale test and intact performance on Digit Span Forward, while one demonstrated deficits in neither. One patient who had lesions to the left inferior and prefrontal cortices had deficits on Digit Span Forward and intact performance on the Scale test. Four out of six patients with injury to right or bilateral frontal regions had deficits on the Rhythm test and intact performance on the Digit Span test, while two patients had deficits in neither (Table 2). 

## 4. Discussion

The present results indicate a high prevalence of music perception deficits resulting from acute TBI, particularly when the damage involves the right hemisphere. Pitch and rhythm deficits often co-occur, but are partially dissociable. Moreover, there is a partial dissociation between domain-general short-term memory and pitch and rhythm perception. This suggests that STM may fractionate into different network dynamics, depending on whether the cognitive task involves the processing of pitch, rhythm, or verbal information. We elaborate on each of these findings below.

### 4.1. Performance on the Scale and Rhythm Subtests of the Montreal Battery of Evaluation of Amusia (MBEA)

In line with our prediction, a significant number of acute TBI patients demonstrated deficits in pitch and rhythm perception. Forty-three percent had pitch deficits, a prevalence ten times greater than in congenital amusia, which Peretz estimates only comprise 4.2% of the general population, when using the more lenient criteria of the Scale test alone [3,27]. Similarly, 40% of the TBI patients demonstrated deficits on the Rhythm test. This was in contrast to age- and education- matched controls, none of whom had pitch deafness, and only one of whom had deficits in rhythm perception. There was no relationship between severity of brain injury and music perception performance, indicating that patients with mild TBI were also vulnerable to deficits in music perception. 

The present results in acute TBI patients expand on results demonstrated in severe chronic TBI, in which pitch and rhythm deficits were identified in 42% and 52% of the patients, respectively [19]. Similar results were also found in a small population of mild chronic TBI patients (*n* =10), who were impaired on the Rhythm test compared to controls, and were marginally outperformed on the Scale test (*p* = 0.07)[18], results that may have achieved significance in a larger sample size. Thus, acquired amusia following TBI may be longstanding based on the results of studies conducted in chronic TBI populations, which were collected between one year and 10 years post-TBI. It would be of interest to perform a longitudinal study comparing short- and long-term deficits in the same patients, in order to determine the recovery rate over time. 

### 4.2. The Relationship of Pitch and Rhythm Processing in Acquired Amusia

As predicted, a significant number of TBI patients demonstrated co-occurring deficits in pitch and rhythm perception. In fact, 31% of acute TBI patients with pitch-deafness demonstrated comorbid rhythm processing deficits (Table 2). This builds on recent results obtained during the chronic phase of TBI, which indicate that 29% of patients have co-occurring pitch and rhythm deficits [19]. Our results are also in keeping with recent findings in congenital amusics that indicate co-occurring pitch and rhythm deficits [43,71]. Finally, co-occurring deficits have been found in patients with acquired amusia following stroke [4,5,10]. The co-occurrence of pitch and rhythm deficits in acute and chronic TBI, stroke, and congenital amusia indicate a close link between pitch and rhythm processing that may point to shared neural networks. However, the co-occurrence of pitch and rhythm deficits may also be the result of communication deficits between separate processing components that contribute to the organization of pitch and temporal information [72].

Given the pitch cues inherent in the Rhythm test of the MBEA, another question arises as to whether pitch processing deficits attenuate rhythm discrimination ability due to simultaneous processing demands, which may erroneously point to shared networks. This is known as the pitch interference hypothesis [43]. A previous study found support for this hypothesis by demonstrating that congenital amusics were able to perceive rhythm when pitch variations were removed [28]. However, the present study does not support the pitch interference hypothesis, as nine patients with impaired pitch perception had spared rhythm perception (Table 2). A recent study in congenital amusics also found that rhythm discrimination was spared in a number of individuals with pitch deafness [43]. Furthermore, when examining beat processing, these difficulties were present even without pitch cues, providing further support for pitch and rhythm deficits as distinct disorders, as beat processing and rhythm processing are closely associated [43]. Finally, the fact that 26% of the TBI patients with music processing deficits demonstrate selective impairment in pitch or rhythm indicates that there is some degree of neural specificity for the treatment of pitch versus rhythm.

Although it is clear that sequential patterns of melody and rhythm are integrated at some level of music processing [8], little is known about the levels at which this occurs. Imaging studies identifying neural correlates for pitch and rhythm processing are sparse. However, one recent study illustrated the close connection between pitch and rhythm processing regions in the brain using voxel-based lesion-symptom mapping (VLSM) [4]. In a sample of 77 stroke patients, deficits on the Scale and Rhythm tests of the MBEA were both associated with right hemisphere lesions in the auditory cortex, Heschl’s gyrus, basal ganglia (putamen, caudate, pallidum), and insula. Furthermore, recovery from acquired amusia was associated with smaller decreases in gray matter in the right temporal lobe for both rhythm and pitch. This indicates shared structures in the processing of rhythm and pitch. However, for pitch amusia, the decreases were observed posteriorly, and for rhythm deficits, anteriorly [5], indicating partial dissociation in the neuroanatomy underlying pitch and rhythm processing. In the present study, a total of 11 patients demonstrated a dissociation between pitch and rhythm processing (nine of whom had impaired pitch and preserved rhythm processing; and two of whom had impaired rhythm and preserved pitch processing).

In sum, the present results in acute TBI patients support a partly shared, partly dissociable network for the processing of pitch and rhythm. This has been proposed in other populations, such as congenital amusia [43], stroke [4,43], and healthy individuals [73]. For example, one study found evidence of anatomically distinct areas in the processing of spectral (tone spacing) versus temporal variations in the auditory cortex of healthy individuals [73]. Future PET and fMRI studies may elucidate the overlap and independence of associated structures and network dynamics. This would expand our understanding of the nature of rhythm processing deficits, and how temporal and melodic dimensions may combine in music processing.

### 4.3. The Lateralization of Pitch and Rhythm Deficits

As predicted, patients with right hemisphere damage had greater deficits in pitch and rhythm perception than those with damage to the left hemisphere. To our knowledge, this has not been previously demonstrated in a group of acute TBI patients. However, the hemispheric lateralization has been demonstrated in other neurological populations, such as stroke [4,35,36], and early single case studies, in which right hemisphere damage was associated with more severe pitch perception deficits in individuals with unilateral temporal lobe excisions [74], temporal lobectomy [75], and stroke [76]. Right hemisphere damage was also associated with difficulty detecting rhythm violations [77] (Fujii, 1990). Finally, our results provide supportive evidence for the predominantly right neural network underlying the processing of pitch and rhythm that has been observed in congenital amusia [11,27,51,78,79]. Both pitch and rhythm activate a network of frontal and temporal areas in the right hemisphere [4].

There is also evidence for interhemispheric involvement in music processing, with left lateralization of certain subsystems [10,11,27,74,80]. Furthermore, acquired amusia is associated with interhemispheric connectivity damage originating in the corpus callosum and tapetum [10]. However, the normal perception of pitch and rhythm depends on functional connectivity between core structures in a distributed network of brain regions located mainly in the right hemisphere. 

This connectivity may be disrupted in TBI patients who suffer from grey matter injury, and/or traumatic axonal injury, which is defined as the stretching, breaking, or twisting of neuron fibres [81]. Damage is commonly located in the frontal lobe, temporal lobe, and corpus callosum, according to studies using diffusion tensor imaging (DTI), a method optimized for detecting white matter injury [81]. White matter tract damage may disrupt fronto-temporal networks connecting auditory regions to inferior frontal regions, which may underlie the maintenance of pitch in STM [27,34,37]. Indeed, in congenital amusics, anomalous processing has been identified in the arcuate fasciculus, a major white matter tract connecting the frontal and temporal regions [27]. Given the fact that the corpus callosum has white matter fibres through which interhemispheric auditory pathways run [82], damage to this region may also contribute to anomalous processing.

Finally, it is possible that the scores of TBI patients in the present study in the context of left or right unilateral lesion do not only reflect an inability of the damaged region to analyze pitch or rhythm information, but also the inhibiting effects of contralateral homologous regions, which may undergo widespread structural changes as an adaptive part of functional reorganization. Support for this phenomenon is found in studies of stroke patients with aphasia [83,84]. Currently, there is a lack of research examining this in TBI patients in the context of music processing, possibly due to the complicating factor of frequently diffuse injuries in this population, and the widespread functional networks involved in music processing. Future studies examining this process in the context of music perception and TBI would be warranted. 

### 4.4. The Relationship between Music Perception Scores and Verbal Short-Term Memory

#### 4.4.1. Pitch Processing and Verbal Short-Term Memory

As hypothesized, a relationship between pitch perception and verbal STM ability was found in 58% of the TBI patients (*n* = 18). These results suggest shared processing components between tonal and verbal material in short-term memory, which is in keeping with research studies conducted in other populations, such as normal healthy individuals [56], musicians [55,57], individuals with congenital amusia [24], and stroke [36]. For example, one study demonstrated that recovery on amusia tests three months post-stroke was associated with the recovery of general-domain verbal memory [6]. 

However, in addition to these shared regions for pitch processing in STM, we provide evidence for at least partly distinct brain mechanisms for pitch and verbal material, as observed in 42% of the TBI patients. The majority of these patients (12/13) had impaired performance at pitch perception (which relies on pitch STM) [1,27] with preserved verbal STM. To our knowledge, this is the first time that this dissociation is being demonstrated in TBI patients with pitch perception deficits. The results of partly shared and partly separate regions for pitch processing are in keeping with results found in stroke patients, some of whom demonstrated impairment only in pitch STM, some only in verbal STM, and some in both [9]. Our results in acute TBI patients expand on studies of individuals with congenital amusia, which have shown that those with a selective impairment in pitch processing have intact verbal STM [22,34,85]. 

To examine the origins of this dissociation, we turn to previous studies examining the contribution of low-level perceptual deficits (such as detection of pitch changes and directions, deviancies, and duration) to amusia. However, these deficits are thought to be a manifestation of amusia, as opposed to its functional root [27,36]. This is based on studies that indicate that while pitch is adequately processed in the amusic brain, it is not done in conscious awareness [86]. Indeed, congenital amusics demonstrate pitch detection thresholds that are comparable to controls, pointing more to a deficit in STM for pitch than low-level perceptual deficits [26]. Furthermore, while certain studies report that pitch detection deficits in amusics decrease performance in tasks measuring pitch STM [1,22,25], these results were based on amusics more accurately discriminating differences in comparison melodies with contour variations or larger pitch intervals, which was similar in controls [26]. Therefore, it is reasonable to propose that the current results of our study point to altered dynamics in the recruitment of higher-order processing, such as pitch STM [21,26], rather than in low-level perceptual pitch deficits.

The altered neural dynamics associated with deficits in short-term memory for pitch are elucidated in a recent fMRI study by Albouy, Peretz, et al. (2019). Compared to controls, congenital amusics demonstrated 1) reduced activity in auditory regions that facilitate low-level sensory processing of fundamental musical elements such as pitch detection; and 2) a deficit in recruiting higher-level association areas, such as the right frontal IFG and DLPFC, key areas for maintaining pitch in short-term memory [34]. A study by Schaal (2015) also identified the role of the DLPFC in maintaining pitch in short-term memory, with findings that transcranial alternating current stimulation applied to the right DLPFC resulted in increased pitch memory in amusics [24]. Reduced connectivity between frontal and temporal regions in the right hemisphere is thought to disrupt feedback/feedforward systems that would normally combine low-level information about pitch and duration [4,8,11,26,74,87] with higher order associative functions, such as short-term memory, awareness, and error correction [27,36,51,79]. Failure to integrate components of elemental musical features with higher-order processing could result in a deficit in an entire domain, such as music, while leaving another domain, such as verbal, intact [27,51].

Indeed, the present study demonstrates that damage to right frontal areas, which are fundamental to pitch processing in short-term memory, may cause deficits in pitch perception, while leaving verbal short-term memory intact. For example, five of six patients with damage involving right frontal regions had impaired pitch perception, and unimpaired verbal STM (Table 2). Based on the aforementioned studies, it is possible that these patients had a diminished capacity for recurrent processing between the right auditory region and the right frontal region due to disrupted connections. This would be expected to compromise pitch STM while leaving verbal STM intact, as the latter relies more heavily on left-lateralized structures [88,89,90,91]. Recent evidence demonstrates that that while verbal and tonal maintenance involves similar structures, network dynamics differ, such that recruitment of these structures is primarily left- lateralized for verbal maintenance, and right-lateralized for tonal maintenance [34]. Damage to these areas is the most significant predictor of severe acquired amusia [5]. Indeed, in the present study we observed that in contrast to the right-lateralized frontal injury accompanying the selective pitch deficit in six patients, there was one patient with lesions in the left inferior and prefrontal cortex who had intact pitch ability, with deficits in verbal STM. 

It should be noted that the present study did not use analogous tasks to measure pitch and verbal STM. However, one study that used analogous tasks in individuals with congenital amusia demonstrated similar results: deficits in pitch STM, with preserved verbal STM [22]. Therefore, there is a clear pattern of dissociation between pitch and verbal STM that mirrors the pattern of that found in congenital amusics. That is, decreased STM for pitch that does not touch STM for verbal information. In sum, the present study results indicate that there are both shared and distinct processing components between verbal and tonal material in STM in TBI patients.

#### 4.4.2. Rhythm Processing and Verbal Short-Term Memory 

As hypothesized, a relationship between rhythm perception and verbal STM was found in 61% of the TBI patients (*n* = 19). Furthermore, similarly to pitch, the results demonstrate a dissociation between performance on rhythm discrimination and verbal STM in 39% of the TBI patients, indicating partly separable subsystems. The majority of these patients (10/12) demonstrated impaired rhythm perception with preserved verbal STM. Scans were available for six of these patients, five of whom also had co-occurring deficits in pitch perception. Four of the six patients demonstrated injury to right or bilateral frontal regions. This was expected, given the extensively overlapping lesion patterns for pitch and rhythm STM located in right frontal, temporal, subcortical structures, and insula [4,42]. However, there are also differences in dynamics and structure between the neural networks treating pitch and rhythm. For example, rhythm deficits are associated with more significant lesions to the right dorsal-striatum than pitch deficits. Furthermore, while recovered pitch amusia is associated with a smaller grey matter volume decrease in the temporoparietal junction, rhythm amusia is associated with a smaller gray matter volume decrease in the inferior temporal pole [5]. 

Finally, it is important to note that in the vast majority of cases of dissociation, verbal STM is preserved in the face of impaired musical STM, whether for pitch or rhythm. Of the individuals with verbal STM deficits, only one of 13 performed adequately on pitch perception, and only two of 12 individuals with verbal STM deficits performed adequately on rhythm perception, providing support for the notion that general-domain STM appears to be a fundamental component to both pitch and rhythm discrimination, and that it fractionates to treat musical versus verbal information.

### 4.5. Implications of Acquired Music Perception Deficits on Music Interventions Following TBI 

Certain studies indicate that more than half of individuals with congenital amusia do not enjoy music [92,93], compared to only 6% of controls [92], or no controls [93]. They incorporated music less frequently into their everyday lives, and experienced fewer mood state changes when listening to music [92,93]. Hyperacousia and other types of auditory dysfunction may occur in certain patients following TBI [94,95,96], and may impact upon these problems. Thus, in TBI patients with music deficits, the goals of music therapy, which include altering mood state, increasing motivation for rehabilitation, and decreasing the stress response [97], may not be met during the acute stage of recovery. 

Thus, given the prevalence of pitch and rhythm disorders in the acute TBI population, and the fact that music listening can significantly contribute to quality of life [98], it is important to include measures of music perception in cognitive follow-up after TBI. This is particularly important in patients with injury involving the right hemisphere, who would be expected to perform most poorly. The MBEA is free of cost, and results may inform patient counseling, and the tailoring of music rehabilitation programs to patients’ individual needs. Screening for music perception would also help identify deficits in patients who are musicians, who may experience a direct impact on employment. 

### 4.6. Limitations

There are several limitations to the present study. First, we did not use audiometry to measure the patients’ hearing ability, due to time constraints in testing. However, patients who reported that they suffered from hearing deficits when questioned were excluded from the study. We also confirmed that the stimuli during the sample trials was audible, and hearing deficits were not noted in conversation. 

Second, we used only the Scale and Rhythm subtests of the MBEA, as testing time in an acute care setting is limited due to patient fatigue, pain, and nausea. These two tests are thought to best represent the melodic and temporal dimension of music perception, with the Scale test identified as the most diagnostic subtest of amusia in the MBEA [27,99,100]. It has been used alone to flag amusia cases in several studies [6,18,99]. Furthermore, the combination of scale and rhythm subtests is thought to provide a reasonable estimate of overall music perception [6]. However, certain researchers have suggested that using the cut-offs for these tests may result in a negatively skewed distribution and over-diagnosis of amusia cases [101,102]. It is important to acknowledge that the MBEA was created for clinical screening, rather than as a stand-alone diagnostic battery. It plays a critical role in the comprehensive evaluation of amusia, which includes tests of audiometry, cognitive assessments, and questionnaires. Nevertheless, this fact would likely not account for the significant number of cases of impaired music perception in the present findings, as a control group was used for comparison. Furthermore, there is abundant evidence for similar patterns of impaired music perception in other neurological populations that support the present findings [4,35,36,74,75].

Third, CT scans, which detect damage on the millimeter (and sometimes sub-millimeter) level, may fail to detect damage in patients with mild brain injury, who often exhibit neuronal damage only at the micron and nanometer level [103,104]. While MRI provides more detailed views than CT, both methods are limited to detecting only the largest lesions [105]. Furthermore, CT and MRI are unable to directly identify injured axons [106,107]. Therefore, it is possible in the present study that even patients with no observable lesions on CT (*n* = 18) had undetected damage that contributed to their music perception deficits. Head CT is used as a screening tool to identify the presence of hemorrhage, rather than whether an individual has a TBI. Future studies using DTI in a larger sample size would allow for more accurate anatomical identification of brain injury. Examining damage to white matter tracts in TBI patients in clinical and research settings might further characterize the connectivity of neural networks involved in music processing. Ideally, longitudinal studies encompassing the acute and chronic phases of TBI would help to formulate more direct conclusions as to the effect of lesion location on music perception. Understanding the role of music perception and processing would enable the development of evidence-based rehabilitation protocols using music therapy in TBI, which is already being used, despite a lack of comprehensive understanding of neural networks. 

Fourth, the fact that there was no relationship between injury severity and performance on the Scale and Rhythm tests may be attributable to the fact that post-traumatic amnesia is more common in TBI patients with severe injury, and precludes them from participating, resulting in an under-representation of this sub-population in this study. 

Fifth, Patients were matched with controls on education and age only, not on sex or years of musical training. Thus, the sex of the individuals in the present study is not equivalent between groups. Evidence for the influence of sex on music cognition is unknown, as studies examining sex differences in music cognition are sparse. However, two studies reported limited, if any significant differences between sexes in performance on tasks of pitch perception. For example, one study demonstrated differences in cerebellar activation patterns between adult males and females on a pitch memory task. However, this did not correspond to differences in scores between groups. Because performance differences could not explain activation differences, the authors concluded that the differing activation pattern between males and females may simply reflect different processing strategies [79]. A second study that examined the ability to distinguish between two tones found that females answered correctly 1% more frequently than males. However, it was unclear as to whether this difference was significant, as statistical analyses were not performed [108]. However, given the small percentage, it is unlikely. As for musical training, this information was not available for controls. However, the majority of TBI patients in the present study had fewer than three years of musical training, with only five patients reporting three or more years (see Table 1). It is unlikely that these five patients would significantly influence the results, given that 24 patients had fewer than three years of musical training. Importantly, the MBEA was created for people without musical training. Thus, the influence of musical training did not likely significantly influence results. 

## 5. Conclusions

We have shown that patients with acute TBI have a high incidence of pitch and rhythm processing deficits that was previously under-recognized in this population. Pitch and rhythm deficits co-occur in TBI patients one third of the time, but occur separately two-thirds of the time. This suggests partly shared but dissociable neural networks for pitch and rhythm. Furthermore, pitch and rhythm processing are predominantly right lateralized, so that traumatic injury to right frontal and temporal areas is associated with these deficits. Finally, neural networks underlying the processing of verbal STM, pitch STM, and rhythm STM intersect, but are partly dissociable. We suggest that general domain STM is recruited and combined with specific areas that process pitch, rhythm, and verbal material. Further studies examining how music-processing deficits relate to other cognitive deficits, especially those involving the right hemisphere, would be useful. Morphometric or functional imaging studies in TBI patients with well-characterized deficits in the musical perception domain may better elucidate the functional and structural connectivity networks that underlie music processing. Longer follow up of these patients may demonstrate to what extent recovery is possible following acute TBI. Finally, evaluation of the frequency and characteristics of musical anhedonia in TBI patients may inform music therapy interventions.

## Figures and Tables

**Table 1:** Patient Demographics and Accident History.

Variable	
Age at injury (*M* ± *SD*)	51.1 ± 18.5
Sex	
Male	36 (86%)
Female	6 (14%)
Musical Training (in years; *M* ± *SD*)	1.2 ± 2.6
<3 years	24 (57%)
≥3 years	5 (12%)
Accident Variables	
TBI Etiology	
Motor vehicle accident	16 (38%)
Fall	15 (35%)
Assault	3 (7%)
Suicide attempt	2 (5%)
Sports	2 (5%)
Other	4 (9%)
TBI Severity	
mild	34 (79%)
moderate	3 (7%)
severe	5 (17%)
LOC	
No	14 (33%)
Yes	28 (67%)
PTA	
No	11(26%)
Yes	31(74%)
LOS (in days; *M* ± *SD*)	14.70 (9.36)
Delay (days from accident to evaluation); M ± SD)	6.7 ± 4.6

Note. LOC = loss of consciousness; PTA = post-traumatic amnesia; LOS = length of stay.

**Table 2:** Individual patient characteristics, performance on the Scale and Rhythm tests of the MBEA, and the Digit Span Forward test of the WAIS-III.

	Patient Characteristics					Tests		
	Age	Gender	Severity	Hemi	Description of Injury	Scale (Z)	Rhythm (Z)	Digit Span Fwd (Z)
P1	23	M	moderate		--	−2.28	−1.62	−0.09
P2	50	M	mild complex		no mass effect	1.25	0.57	−0.69
P3	63	F	mild		--	−6.40	−1.62	−2.08
P4	65	M	moderate	B	left holohemispheric SDH (20 mm); midline shift 10 mm; previous surgical resection of right temporal lobe in 1967.	−4.64	−2.93	--
P5	61	M	mild		no acute intracranial findings	−0.52	−1.62	--
P6	35	M	moderate	B	multiple small foci of hemorrhagic contusions in the white matter of pre-SMA and SMA of frontal lobes	−2.87	−2.93	−1.50
P7	37	M	mild		no acute intracranial findings	−1.69	−3.81	−1.50
P8	64	M	mild complex		no mass effect	0.66	0.57	−0.54
P9	62	M	mild		--	1.25	−0.74	--
P10	62	M	severe	L	holohemispheric SDH (27 mm), midline shift 6 mm left to right	0.07	0.14	0.23
P11	40	M	mild		no acute intracranial findings	−1.11	−1.62	--
P12	78	F	moderate	R	frontal lobe intraparenchymal hematoma (32 mm)	−6.99	−3.37	−1.00
P13	63	M	mild complex	B	SDH in right parietal lobe (15 mm) and left frontal lobe (13 mm).	−5.22	−2.06	--
P14	43	M	mild complex		no mass effect	0.66	0.57	0.17
P15	72	M	mild complex	R	hemorrhagic contusion in the pre-SMA of the right superior frontal gyrus (12 mm)	−3.46	−3.81	--
P16	17	M	mild complex		no mass effect	−4.05	−0.74	--
P17	73	M	mild complex		no mass effect	−2.87	−0.30	--
P18	55	M	mild complex		no mass effect	−3.46	−2.06	−1.31
P19	52	M	mild complex	R	right parietal (4 mm) and right temporal (3 mm) SDHs	−0.52	−1.62	−1.46
P20	71	M	moderate	R	frontal SDH (10 mm) and residual hypodensities in the temporal lobe, following evacuation for a holohemispheric SDH	−4.64	−3.37	−0.64
P21	43	M	severe	B	multiple small foci of hemorrhagic contusions involving the subcortical white matter of both frontal lobes	−2.28	−2.06	−0.67
P22	55	M	mild complex	L	hemorrhagic contusion in the parahippocampal formation (6 mm).	0.07	−2.50	−1.31
P23	44	M	mild		no acute intracranial findings	0.07	0.14	−1.50
P24	56	F	mild complex	L	hemorrhagic contusions in the inferior frontal and prefrontal cortex	0.07	0.57	−2.08
P25	60	M	mild complex	L	hemorrhagic contusion in the left frontoparietal area (8 mm)	1.25	−1.18	−0.54
P26	27	F	mild		--	−3.46	−1.18	--
P27	51	M	moderate	L	holospheric hematoma (9 mm) with left to right midline shift (3 mm)	−0.52	−1.18	−0.69
P28	58	M	moderate	R	hemorrhage in the pallidum and putamen (15 mm).	−6.99	−3.37	−1.31
P29	48	M	severe		--	−4.64	−2.06	−1.46
P30	87	F	mild complex		no mass effect	−5.22	−2.93	--
P31	33	F	mild		no acute intracranial findings	−3.46	−4.25	--
P32	18	F	mild		no acute intracranial findings	−5.81	−3.37	−2.18
P33	21	M	mild complex		no mass effect	−0.52	−2.50	−1.00
P34	26	M	mild		no acute intracranial findings	0.66	0.14	0.00
P35	20	F	mild		--	1.25	1.01	−1.00
P36	34	F	mild		no acute intracranial findings	−0.52	1.01	1.43
P37	65	M	mild complex		no mass effect	−4.05	−1.18	−0.57
P38	77	M	moderate	L	large holohemispheric mixed density subdural hematoma (13 mm ) with left to right midline shift (9 mm)	−4.64	−1.18	−1.91
P39	56	M	mild complex	B	small frontal hemorrhagic contusions in the SMA (6 mm on right, and 7 mm on left)	−4.64	−0.30	−1.31
P40	52	M	mild complex	R	holohemispheric SDH (18 mm), right to left midline shift (7 mm)	0.07	−0.74	0.08
P41	84	M	mild complex		no mass effect	−5.81	−1.62	−0.85
P42	45	M	mild complex	L	inferior frontal SDH (4 mm)	−0.52	−0.74	−0.69

Note. P = patient; Age in years; M = male; F = female; L=left, R = right, B = bilateral; SDH = subdural hematoma, SMA= supplementary motor area.

## Data Availability

The MBEA database can be found at the Isabelle Peretz Research Laboratory website https://www.peretzlab.ca/publications/2003/page2 (accessed on 25 March 2020). Due to the sensitive nature of patient charts, study participants were assured that raw data would remain confidential and would not be shared.
